# High Rate of HIV Resuppression After Viral Failure on First-line Antiretroviral Therapy in the Absence of Switch to Second-line Therapy

**DOI:** 10.1093/cid/cit933

**Published:** 2013-12-18

**Authors:** Ravindra K. Gupta, Ruth L. Goodall, Michael Ranopa, Cissy Kityo, Paula Munderi, Fred Lyagoba, Lincoln Mugarura, Charles F. Gilks, Pontiano Kaleebu, Deenan Pillay, for the DART Virology Group and Trial Team, H. Grosskurth, P. Munderi, G. Kabuye, D. Nsibambi, R. Kasirye, E. Zalwango, M. Nakazibwe, B. Kikaire, G. Nassuna, R. Massa, K. Fadhiru, M. Namyalo, A. Zalwango, L. Generous, P. Khauka, N. Rutikarayo, W. Nakahima, A. Mugisha, J. Todd, J. Levin, S. Muyingo, A. Ruberantwari, P. Kaleebu, D. Yirrell, N. Ndembi, F. Lyagoba, P. Hughes, M. Aber, A. Medina Lara, S. Foster, J. Amurwon, P. Mugyenyi, C. Kityo, F. Ssali, D. Tumukunde, T. Otim, J. Kabanda, H. Musana, J. Akao, H. Kyomugisha, A. Byamukama, J. Sabiiti, J. Komugyena, P. Wavamunno, S. Mukiibi, A. Drasiku, R. Byaruhanga, O. Labeja, P. Katundu, S. Tugume, P. Awio, A. Namazzi, T. G. Bakeinyaga, H. Katabira, D. Abaine, J. Tukamushaba, W. Anywar, W. Ojiambo, E. Angweng, S. Murungi, W. Haguma, S. Atwiine, J. Kigozi, A. Latif, J. Hakim, V. Robertson, A. Reid, E. Chidziva, R. Bulaya-Tembo, G. Musoro, F. Taziwa, C. Chimbetete, L. Chakonza, A. Mawora, C. Muvirimi, G. Tinago, P. Svovanapasis, M. Simango, O. Chirema, J. Machingura, S. Mutsai, M. Phiri, T. Bafana, M. Chirara, L. Muchabaiwa, M. Muzambi, E. Katabira, A. Ronald, A. Kambungu, F. Lutwama, A. Nanfuka, J. Walusimbi, E. Nabankema, R. Nalumenya, T. Namuli, R. Kulume, I. Namata, L. Nyachwo, A. Florence, A. Kusiima, E. Lubwama, R. Nairuba, F. Oketta, E. Buluma, R. Waita, H. Ojiambo, F. Sadik, J. Wanyama, P. Nabongo, R. Ochai, D. Muhweezi, C. Gilks, K. Boocock, C. Puddephatt, D. Winogron, J. Bohannon, J. Darbyshire, M. D. Gibb, A. Burke, D. Bray, A. Babiker, S. A. Walker, H. Wilkes, M. Rauchenberger, S. Sheehan, L. Peto, K. Taylor, M. Spyer, A. Ferrier, B. Naidoo, D. Dunn, R. Goodall, R. Nanfuka, C. Mufuka-Kapuya, P. Kaleebu, D. Pillay, P. Awio, M. Chirara, D. Dunn, C. Gilks, R. Goodall, A. Kapaata, M. Katuramur, F. Lyagoba, R. Magala, B. Magambo, K. Mataruka, A. McCormick, L. Mugarura, T. Musunga, M. Nabankkema, J. Nkalubo, P. Nkurunziza, C. Parry, V. Robertson, M. Spyer, D. Yirrell, A. Medina Lara, S. Foster, J. Amurwon, B. Nyanzi Wakholi, J. Kigozi, L. Muchabaiwa, M. Muzambi, I. Weller, A. Babiker, S. Bahendeka, M. Bassett, A. Chogo Wapakhabulo, J. Darbyshire, B. Gazzard, C. Gilks, J. Hakim, A. Latif, C. Mapuchere, O. Mugurungi, P. Mugyenyi, C. Burke, S. Jones, C. Newland, S. Rahim, J. Rooney, M. Smith, W. Snowden, J.-M. Steens, A. Breckenridge, A. McLaren, C. Hill, J. Matenga, A. Pozniak, D. Serwadda, T. Peto, A. Palfreeman, M. Borok

**Affiliations:** 1 Department of Infection; 2 Medical Research Council (MRC) Clinical Trials Unit, University College London, United Kingdom; 3 Joint Clinical Research Centre, Kampala; 4 MRC/Uganda Virus Research Institute, Uganda Research Unit on AIDS, Entebbe, Uganda; 5 School of Population Health, University of Queensland, Brisbane, Australia; 6 Wellcome Trust Africa Centre for Health and Population Sciences, University of KwaZulu Natal, Mtubatuba, South Africa

**Keywords:** HIV, failure, viral resuppression, resistance, Africa

## Abstract

In a randomized comparison of nevirapine or abacavir with zidovudine plus lamivudine, routine viral load monitoring was not performed, yet 27% of individuals with viral failure at week 48 experienced resuppression by week 96 without switching. This supports World Health Organization recommendations that suspected viral failure should trigger adherence counseling and repeat measurement before a treatment switch is considered.

Combination antiretroviral therapy (cART) has led to declining morbidity and mortality in resource-poor settings [1, 2], and scale-up at the end of 2012 had reached 9.7 million human immunodeficiency virus (HIV)–infected individuals worldwide [3]. Optimal utilization of first-line cART and switch to second-line therapy in resource-poor settings is a priority. World Health Organization (WHO) guidelines recommend routine viral load monitoring (VLM), and switch to second-line therapy is recommended after 2 viral load measurements >1000 copies/mL following adherence counseling [4]. However, the WHO document recognizes that the evidence base for VLM itself is weak. Given that such a monitoring strategy is likely to be a huge burden for most resource-limited settings, it is important to increase the evidence base. Furthermore, there is a limited body of data on how viremia evolves on therapy in absence of VLM, and the impact on emergence of drug resistance; such information is needed to inform treatment guidelines.

The Development of Anti Retroviral Therapy in Africa (DART) study compared clinical monitoring only with clinical and laboratory monitoring (CD4 and routine blood tests including biochemistry and full blood count), with switch to second-line therapy on clinical and immunologic criteria. This study demonstrated good clinical outcomes in both arms over the 5-year follow-up period [5]. In a substudy of DART, a comparison of zidovudine (ZDV)/lamivudine (3TC)/nevirapine (NVP) vs ZDV/3TC/abacavir (ABC) (NORA Study) showed triple nucleosides (ZDV/3TC/ABC) to be associated with higher rates of virologic and immunologic failure than the nonnucleoside reverse transcriptase inhibitor (NNRTI)–based regimen (ZDV/3TC/NVP) at 48 weeks [6, 7]. Here we report virologic outcomes at 96 weeks, demonstrating substantial resuppression following earlier viremia despite not switching.

## METHODS

The NORA Study enrolled 600 previously untreated and asymptomatic Ugandan participants with CD4 counts of <200 cells/µL, randomly assigned to coformulated ZDV/ 3TC and either ABC and NVP placebo (ABC arm), or ABC placebo and NVP (NVP arm). Each drug was taken twice daily. After 24 weeks, participants continued to receive the study drugs open label and were followed as part of DART for a minimum of 4 years. In a separate randomized substudy, participants with a CD4 count ≥300 cells/µL at 48 or 72 weeks after ART initiation were eligible to be randomized to continuous therapy or structured treatment interruption (STI) with repeated 12-week periods on or off therapy [8]. Viral loads were retrospectively measured using Roche Amplicor 1.5.

Analyses were based on participants who were alive, in follow-up, and still on first-line therapy at week 96, and who were not randomized to the STI arm in the STI substudy. Although the latter exclusion was essential because of the effect of STIs on viral load (and also possibly development of drug resistance), it introduces a different bias as eligibility for the STI substudy was related to earlier viral load values via the CD4 count inclusion criterion. The effect of this is the selective exclusion of participants with a good early virologic response and therefore, in crude analyses, underestimation of the rate of viral suppression at week 96. To account for this bias, inverse probability weights (separate for the 2 NORA arms) were used to up-weight participants who were randomized to continuous therapy.

Week 96 samples with a viral load >1000 copies/mL underwent resistance testing by standard population sequencing of *pol* [6]. The frequencies of resistance-associated mutations [9] were calculated both for all participants (intention-to-treat) and for participants who had made no major substitutions (defined in the Results section) to their initial regimen (on-treatment). Participants with baseline resistance were excluded from analyses of resistance.

Ethics approval both for DART and the NORA substudy was obtained both in Uganda (Uganda Research Unit on AIDS Science and Ethics Committee) and the United Kingdom (Imperial College).

## RESULTS

Of the 600 participants randomized in NORA (300 ABC arm, 300 NVP arm), 32 died before week 96 (13 ABC, 19 NVP), 21 were lost to follow-up (10 ABC, 11 NVP), and 107 were randomized to structured treatment interruption (37 ABC, 70 NVP). Seven participants (4 ABC, 3 NVP) switched to a second-line regimen based on lopinavir/ritonavir after week 48 and are excluded from all analyses; all achieved virologic suppression by 96 weeks. The number left for evaluation at week 96 was 236 and 197 in the ABC and NVP arms, respectively (Supplementary Figure 1). Twenty-five (11%) participants made a substitution in the ABC arm (from ABC to NVP or tenofovir [TDF]) and 28 (14%) in the NVP arm (from NVP to ABC or TDF).

Consistent with previously reported week 48 data [6], the distribution of viral load at week 96 differed between the 2 arms (*P* < .001; Table 1), with a greater proportion of participants in the NVP arm achieving viral load suppression <1000 copies/mL. The viral load in the majority of participants with suppression was <200 copies/mL in both arms (91% and 95% of those <1000 copies/mL in the ABC and NVP arms, respectively). Table 1 shows the association between viral load at week 48 and week 96 for individual participants. Participants with viral load <1000 copies/mL at week 48 were likely to remain <1000 copies/mL at 96 weeks, although more so in the NVP arm (96% [149/156]) than in the ABC arm (82% [148/180]) (*P* = .003).

**Table 1.** Distribution of Week 96 HIV RNA Viral Load by Week 48 HIV RNA and Treatment Arm

Viral Load	ABC					NVP				
	Week 96 VL, Copies/mL					Week 96 VL, Copies/mL				
	<1000	1000–9999	10 000–99 999	≥100 000	Total	<1000	1000–9999	10 000–99 999	≥100 000	Total
Week 48 VL, copies/mL										
<1000	148	16	12	4	180	149	2	5	0	156
1000–9999	9	7	5	1	22	0	3	1	0	4
10 000–99 999	1	1	6	6	14	4	1	6	1	12
≥100 000	2	0	4	4	10	3	0	2	3	8
Total	160	24	27	15	226	156	6	14	4	180
Adjusted total, % (95% CI)	71.1 (65.4–76.1)	11.6 (7.9–16.7)	11.5 (8.1–16.1)	5.8 (3.8–8.9)		88.7 (84.6–91.9)	3.6 (1.6–8.0)	5.9 (3.8–9.0)	1.7 (0.8–3.9)	

Adjusted percentages were calculated using inverse probability weights to account for missing values due to structured treatment interruption randomization (37 ABC, 70 NVP) or missing sample/failed assay (9 ABC, 11 NVP). Participants who died, were lost to follow-up, or started second-line treatment before week 96 were excluded (27 ABC, 33 NVP), as were 7 (1 ABC, 6 NVP) participants with no week 48 VL.

Abbreviations: ABC, abacavir; CI, confidence interval; NVP, nevirapine; VL, viral load.

Nineteen of 70 (27%) of individuals (12/46 ABC vs 7/24 NVP; *P* = .82) with viral load >1000 copies/mL at week 48 experienced resuppression by week 96, indicating issues with adherence. Sixty-seven of these 70 patients had drug resistance data at week 48; 10 of 57 (18%) individuals with at least 1 major mutation at week 48 had experienced resuppression by 96 weeks. Resistance patterns present in these 10 individuals were M184V (n = 3), M184V + D67N, M184V + T215Y, M184V + Y181C, M184V + D67N + K70R (n = 3), and Y188C. Among the remaining 10 individuals who had no resistance mutations at week 48, 7 (70%) were resuppressed, suggesting an improvement in adherence after week 48. Two of 3 individuals with no resistance result available at week 48 experienced resuppression by week 96.

Of 91 participants with viral load ≥1000 copies/mL) at week 96, 87 (96%) had a genotype available. Five (4 ABC, 1 NVP) participants with baseline resistance were excluded, leaving 82 (59 ABC, 23 NVP) patients for analysis. The frequencies of mutations for both the intention-to-treat and on-treatment populations are given in Supplementary Table 2. The following description focuses on the on-treatment population for simplicity. A high proportion of failures in the NVP arm had major NNRTI resistance at week 96 (95%). Thirteen (68%) had only 1 NNRTI mutation, and 5 (26%) participants had 2 NNRTI mutations. The M184V mutation, conferring resistance to 3TC, was highly prevalent (90% ABC, 89% NVP). The proportion of participants with ≥3 thymidine analogue mutations (TAMs) at week 96 was similar between the ABC group (49%) and the NVP group (42%) (*P* = .79). In the former group, ABC-specific mutations L74V and K65R were each seen in 1 individual. The pan–nucleoside resistance mutation Q151M was not observed in any individual.

## DISCUSSION

We present 2-year virologic data from the DART-NORA study, highlighting the very good suppression rates achieved using ZDV/3TC and NVP. Viral failure as defined by WHO [4] was almost 3-fold higher with triple nucleoside reverse transcriptase inhibitors (NRTIs) containing ZDV/3TC/ABC compared to that seen in ZDV/3TC/NVP–treated individuals, and supports the recommendation that this combination not be used for first-line therapy in adults when alternative drugs are available. There was a high prevalence of extensive NRTI cross-resistance following viral failure at week 96, with almost half of patients in each treatment arm having ≥3 TAMs, consistent with other studies in resource-limited settings [10, 11]. Nonetheless, in vivo residual activity of approximately 1 log in viral load was observed in both NORA treatment groups overall [12]. The residual activity in the NVP group was lower than the triple NRTI group, consistent with NNRTI mutations conferring high-level resistance [13].

We noted that most individuals treated with NVP who developed NNRTI resistance had a single mutation only, consistent with previous reports examining viral failure with both efavirenz- and NVP-containing regimens [14–17]. This questions the assumption that prolonged viral failure necessarily leads to accumulation of NNRTI mutations.

Most importantly, we found that one-quarter of individuals with viral failure (>1000 copies/mL) at week 48 experienced resuppression at 96 weeks even though real-time viral load testing was not undertaken. This was most likely due to an improvement in adherence. It is notable that resuppression occurred in the presence of major resistance mutation(s) at week 48 and no change in therapy, suggesting that strong antiviral activity is possible despite reduced viral susceptibility, although the role of adherence cannot be ignored. Drug substitutions due to poor tolerability/side effects did not account for the observed changes in viral load. In South Africa, where real-time viral monitoring has taken place, substantial rates of resuppression without modification of ART have also been reported, even in patients with NNRTI resistance [18]. Where VLM is introduced more widely, our data support WHO recommendations that suspected viral failure should be addressed by adherence counseling as well as repeat measurement before consideration of treatment switch. Such counseling might identify specific issues with the regimen and culminate in a treatment substitution to achieve a better fit for the patient and therefore better adherence.

## Supplementary Data

Supplementary materials are available at *Clinical Infectious Diseases* online (http://cid.oxfordjournals.org/). Supplementary materials consist of data provided by the author that are published to benefit the reader. The posted materials are not copyedited. The contents of all supplementary data are the sole responsibility of the authors. Questions or messages regarding errors should be addressed to the author.

Supplementary Data
