# Supplementary material for: High Rate of HIV Resuppression After Viral Failure on First-line Antiretroviral Therapy in the Absence of Switch to Second-line Therapy
Source: Clin Infect Dis. 2013 Dec 18;58(7):1023–6. doi: 10.1093/cid/cit933 (PMC3952602; doi:10.1093/cid/cit933)
Supplement: Supplementary Data [file supp_58_7_1023__index.html]

High Rate of HIV Resuppression After Viral Failure on First-line Antiretroviral Therapy in the Absence of Switch to Second-line Therapy — High Rate of HIV Resuppression After Viral Failure on First-line Antiretroviral Therapy in the Absence of Switch to Second-line Therapy — Supplementary Data 

# High Rate of HIV Resuppression After Viral Failure on First-line Antiretroviral Therapy in the Absence of Switch to Second-line Therapy

## Supplementary Data

Supplementary Data

**Files in this Data Supplement:**

- Supplementary Data - Docx file
