# Supplementary material for: High Rate of HIV Resuppression After Viral Failure on First-line Antiretroviral Therapy in the Absence of Switch to Second-line Therapy
Source: Clin Infect Dis. 2013 Dec 18;58(7):1023–6. doi: 10.1093/cid/cit933 (PMC3952602; doi:10.1093/cid/cit933)

**Supplementary Figure 1:** Flow chart for DART-NORA participants.

**Supplementary Table 1:** Resistance mutations at week 96. Numbers of individuals by treatment arm are shown with percentage in brackets. ABC- abacavir; NVP – nevirapine; ^a^ ITT = Intention To Treat population (all participants) ^b^ OT = On Treatment population – excluding 12 participants with major substitutions to initial regimen: 8 ABC arm (from ABC to NVP or TDF) and 4 NVP arm (from NVP to ABC or TDF).


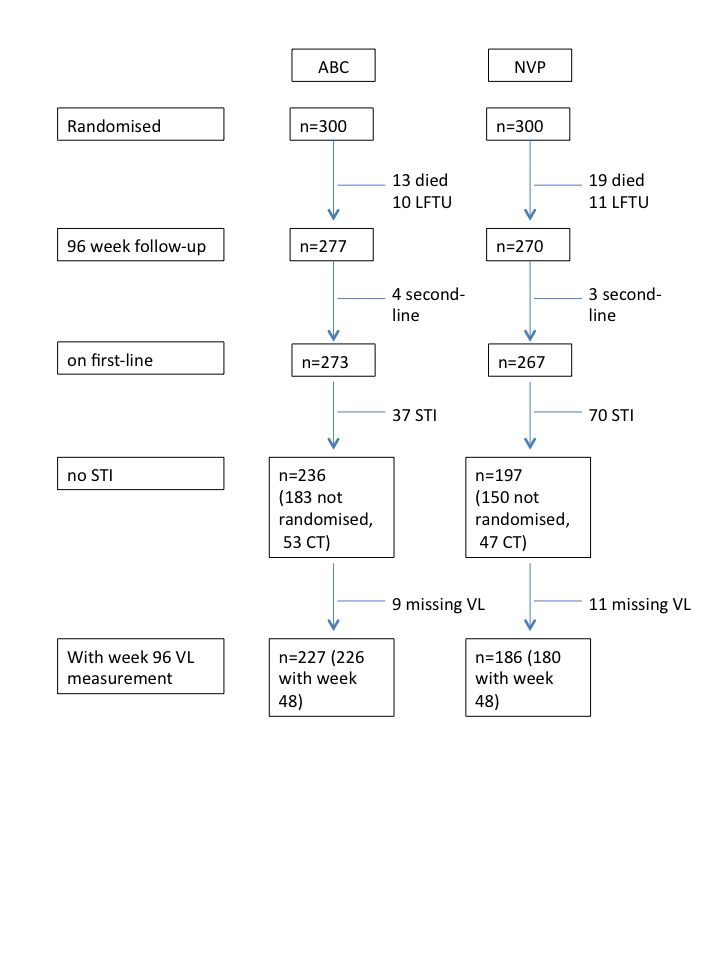

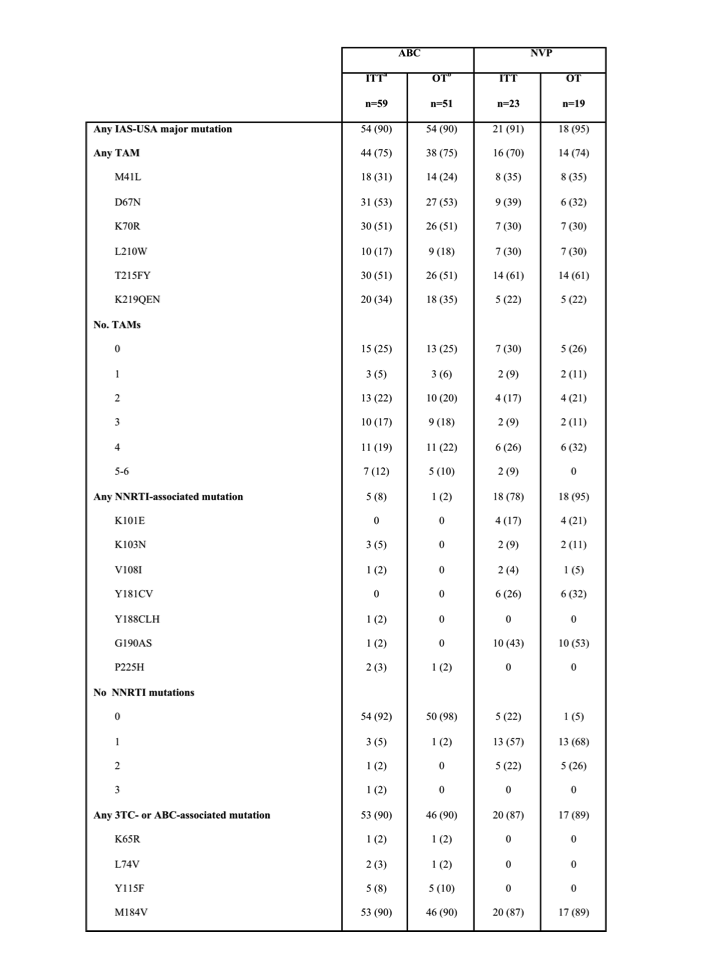

Supplement: Supplementary Data [file supp_cit933_cit933supp.docx]
